# Complete Sequencing of the *bla*_NDM-1_-Positive IncA/C Plasmid from *Escherichia coli* ST38 Isolate Suggests a Possible Origin from Plant Pathogens

**DOI:** 10.1371/journal.pone.0025334

**Published:** 2011-09-23

**Authors:** Tsuyoshi Sekizuka, Mari Matsui, Kunikazu Yamane, Fumihiko Takeuchi, Makoto Ohnishi, Akira Hishinuma, Yoshichika Arakawa, Makoto Kuroda

**Affiliations:** 1 Laboratory of Bacterial Genomics, Pathogen Genomics Center, National Institute of Infectious Diseases, Tokyo, Japan; 2 Department of Bacteriology II, National Institute of Infectious Diseases, Tokyo, Japan; 3 Department of Bacteriology, National Institute of Infectious Diseases, Tokyo, Japan; 4 Deparment of Infection Control and Clinical Laboratory Medicine, Dokkyo Medical University, Mibu, Tochigi, Japan; 5 Department of Bacteriology, Nagoya University Graduate School of Medicine, Nagoya, Japan; University of Hyderabad, India

## Abstract

The complete sequence of the plasmid pNDM-1_Dok01 carrying New Delhi metallo-β-lactamase (NDM-1) was determined by whole genome shotgun sequencing using *Escherichia coli* strain NDM-1_Dok01 (multilocus sequence typing type: ST38) and the transconjugant *E. coli* DH10B. The plasmid is an IncA/C incompatibility type composed of 225 predicted coding sequences in 195.5 kb and partially shares a sequence with *bla*_CMY-2_-positive IncA/C plasmids such as *E. coli* AR060302 pAR060302 (166.5 kb) and *Salmonella enterica* serovar Newport pSN254 (176.4 kb). The *bla*_NDM-1_ gene in pNDM-1_Dok01 is terminally flanked by two IS*903* elements that are distinct from those of the other characterized NDM-1 plasmids, suggesting that the *bla*_NDM-1_ gene has been broadly transposed, together with various mobile elements, as a cassette gene. The chaperonin *groES* and *groEL* genes were identified in the *bla*_NDM-1_-related composite transposon, and phylogenetic analysis and guanine-cytosine content (GC) percentage showed similarities to the homologs of plant pathogens such as *Pseudoxanthomonas* and *Xanthomonas* spp., implying that plant pathogens are the potential source of the *bla*_NDM-1_ gene. The complete sequence of pNDM-1_Dok01 suggests that the *bla*_NDM-1_ gene was acquired by a novel composite transposon on an extensively disseminated IncA/C plasmid and transferred to the *E. coli* ST38 isolate.

## Introduction

Gram-negative bacteria have acquired mobile genetic elements associated with multiple resistance determinants for most antibiotic classes. Six ESKAPE pathogens (***E*** *nterococcus faecium*, ***S*** *taphylococcus aureus*, ***K*** *lebsiella pneumoniae*, ***A*** *cinetobacter baumannii*, ***P*** *seudomonas aeruginosa*, and ***E*** *nterobacter* spp.) are currently recognized as some of the most problematic bacterial challenges facing the infectious disease community [1]. In Gram-negative bacteria, the most common β-lactam resistance mechanism involves β-lactamase-mediated hydrolysis, which leads to inactivation of antibiotics [2]. Metallo-β-lactamase (MBL) genes, which hydrolyze all β-lactams including carbapenems (except aztreonam), are increasing in frequency among Gram-negative organisms such as multidrug-resistant *Enterobacteriaceae* [3]. In 2008, a novel MBL, New Delhi metallo-β-lactamase (NDM-1), was identified in *K. pneumoniae* (strain 05-506) and *Escherichia coli* isolates from a Swedish patient who was transferred from India [4].

There is growing concern about the global emergence of NDM-1-positive bacteria [5], [6], and the first Japanese case of NDM-1-positive *E. coli* (strain NDM-1_DOk01) was a Japanese man who traveled to India in March 2009 [7]. Further dissemination of NDM-1 is of concern due to the identification of NDM-1-positive organisms in waste seepage and tap water in New Delhi [8]. To complicate matters, NDM-1 has been identified in virulent bacteria such as *Vibrio cholera* and *Shigella* spp. [8]. A recent surveillance study showed that NDM-1-positive isolates were circulating in New Delhi as early as 2006, and it was two years before the first European case was reported in 2008 [9].

Such dissemination and wide transmission of NDM-1 among *Enterobacteriaceae* is of great concern. Transfer of NDM-1-encoding plasmids occurs in a temperature-dependent manner, with higher rates of transfer at 30°C compared with 25°C or 37°C [8]. This finding suggests serious implications for the environmental transfer of NDM-1 because the average daily peak temperature in New Delhi reaches 30°C in 7 months of the year (April–October) [8]. Furthermore, additional genetic information is required to characterize the transmission events [10]. NDM-1 was originally found on a plasmid of ∼180 kb, but the incompatibility group (Inc) could not be defined [4]. A subsequent study identified NDM-1 on plasmids of various sizes (∼50–300 kb) that belonged to different Inc groups, including A/C, FI/FII, and an untyped group [11]. The IncA/C plasmid has been identified in *E. coli*, *Citrobacter freundii*, and *Vibrio cholerae* isolates from New Delhi waste seepage [8]. The first complete sequence of an IncL/M pNDM-HK plasmid encoding NDM-1 has already been reported [12]. Here, we report the complete sequence of the IncA/C pNDM-1_Dok01 plasmid carrying NDM-1 in an *E. coli* NDM-1_Dok01 strain, which was isolated from the first case in Japan.

## Methods

### Bacterial strains

The NDM-1-producing *E. coli* strain NDM-1_Dok01 was isolated from the first reported case in Japan [7]. The NDM-1 plasmid was transferred to the streptomycin-resistant *E. coli* DH10B strain via conjugation and maintained by selection with 800 µg/mL streptomycin and 16 µg/mL ceftazidime.

### Short-read DNA sequencing

Two *E. coli* NDM-1_Dok01 strain DNA libraries (∼600 bp and 1.3 kb) were prepared using the Genomic DNA Sample Prep Kit (Illumina, San Diego, CA). DNA clusters were generated on a slide using the Cluster Generation Kit (ver. 4) on an Illumina Cluster Station (Illumina) according to the manufacturer's instructions. In addition, a plasmid that was transferred from NDM-1_Dok01 to the DH10B strain was also sequenced as described above. All sequencing runs for 70 mers were performed using an Illumina Genome Analyzer IIx (GA IIx) with the TruSeq SBS Kit v5. Fluorescent images were analyzed using the Illumina RTA1.8/SCS2.8 base-calling pipeline to obtain FASTQ-formatted sequence data.

### *De novo* assembly of short DNA reads and gap-closing

Prior to *de novo* assembly, the obtained 70-mer reads were assembled using ABySS-pe v1.2.5 [13] with the following parameters: j2, k50, n30, c44.8636, t10, and q40. Predicted gaps were amplified with a specific PCR primer pair, followed by Sanger DNA sequencing with the BigDye Terminator v3.1 Cycle Sequencing Kit (Applied Biosystems, Foster City, CA).

### Validation of gap closing and sequencing errors by short-read mapping

To validate whether mis-assembled sequences and incorrect gap-closing remained after reference-assisted gap-closing, 40-mer short reads were aligned to the tentative complete plasmid DNA sequence using Maq software (ver. 0.7.1) with the *easyrun* Perl-command [14]. We then performed a read alignment to validate possible errors using the MapView graphical alignment viewer [15].

### Annotation

Gene prediction was performed for the complete plasmid sequence with GeneMarkS and followed by GeneMark.hmm prokaryotic version 2.6p [16]. A BLASTP homology search was performed for product assignment. Genomic information, such as nucleic variations and circular representations, was analyzed with IMC-GE software (in silico biology Inc., Yokohama, Japan).

### Multilocus sequence typing

The sequence type (ST) of the *E. coli* isolate was determined on the Multilocus sequence typing (MLST) website (http://mlst.ucc.ie/mlst/dbs/Ecoli) using the predicted coding sequence from *de novo* assemblies.

### Pairwise alignment of plasmids

Pairwise alignment was performed by a BLASTN homology search [17] between the elements, followed by visualization of the aligned images with the ACT program [18].

### Phylogenetic analysis

All amino acid sequences were aligned with clustalW, followed by phylogenetic analysis using the maximum likelihood method with 1,000-times bootstrapping in MEGA5 software [19]. FigTree ver. 1.2.3 software was used to display the generated tree.

### Nucleotide sequence accession numbers

The complete sequence of pNDM-1_Dok01 has been deposited into the DNA Data Bank of Japan (DDBJ; accession number: AP012208).

## Results

### Complete sequence of pNDM-1_Dok01 in *E. coli* NDM-1_Dok01

**Figure 1:**
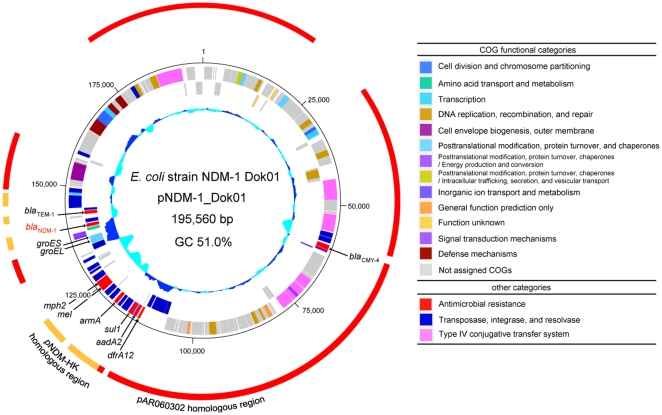
Circular representation of the *E. coli* NDM-1_Dok01 plasmid pNDM-1_Dok01. From the outside inwards, the outer circle indicates the homologous regions to the *E. coli* strain AR060302 plasmid pAR060302 (red) and *E. coli* strain HK-01 plasmid pNDM-HK (orange). The second circle shows the size in base pairs (bp). The third and fourth circles show the positions of the CDSs transcribed in the clockwise and anti-clockwise directions, respectively (using color codes according to the clusters of orthologous groups (COG) classification table and additional customized categories). The fifth circle shows a plot of the G + C content (in 0.5 kb windows).

The complete sequence of pNDM-1_Dok01, carrying the *bla*_NDM-1_ gene, was determined from the genomic DNA of the *E. coli* NDM-1_Dok01 strain by *de novo* shotgun sequencing, assembly, and gap-closing. *De novo* shotgun sequencing of the transconjugant DH10B strain, which harbors the plasmid transferred by filter-mating conjugation, was performed and revealed the plasmid to be composed of 225 predicted coding sequences (CDSs) of 195,560 bp with a guanine-cytosine content (GC) of 51.0% (Fig. 1).

The whole plasmid partially shared the sequence with the *bla*_CMY-2_-positive IncA/C pAR060302 plasmid (166.5 kb) in *E. coli* AR060302 and pSN254 (176.4 kb) in *Salmonella enterica* serovar Newport [20]. The IncA/C incompatibility group of pNDM-1_Dok01 can be determined by *in silico* polymerase chain reaction (PCR) using the PCR-based replicon typing (PBRT) primers described by Carattoli *et al.* [21]; however, the primer A/C-RV sequence has 2 nucleotide mismatches with the corresponding sequence in pNDM-1_Dok01, suggesting that the PCR assay might fail due to such variation in primer sequence. These plasmids share the same type of replicon, type IV conjugative transfer machinery (*tra*), *bla*_CMY-4_ gene, and class I integron, except for the variable region around the *bla*_NDM-1_ gene (Fig. 1).

**Figure 2:**
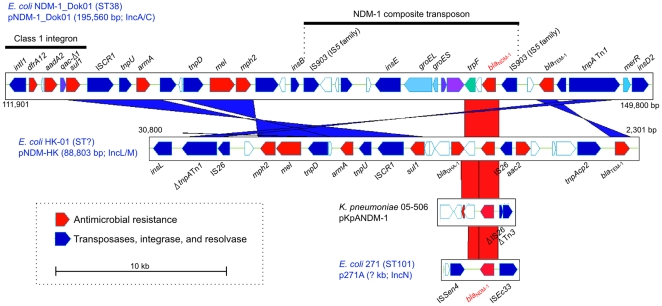
Schematic representation of multiple drug-resistance determinants. Pairwise comparison of plasmid regions around the *bla*_NDM-1_ gene in pNDM-1_Dok01, pNDM-HK, and pKpANDM-1 in *K. pneumoniae* KP-05-506 and *E. coli* strain 271 by a BLASTN homology search and visualized with the ACT program. The *bla*_NDM-1_ genes are identical among the aligned sequences. The red and blue bars between the DNA represent individual nucleotide matches in the forward and inverted directions, respectively. BLASTN match scores of <300 are not shown.

The complete sequence of the NDM-1 pNDM-HK plasmid (88.8 kb) [12] possesses an IncL/M incompatibility group, and similar antibiotic resistance markers (*sul1, armA, macB, mph2, bla*_NDM-1_, and *bla*_TEM-1_) to those of pNDM-1_Dok01 in the present study. Although these antibiotic resistance markers appeared to be shared between pNDM-HK and pNDM-1_Dok01 (Fig. 1), pairwise alignment between the two plasmids showed completely different gene organization (Fig. 2).

### Comparison of gene organization around the *bla*_NDM-1_ gene between plasmids

Surprisingly, the flanking IS elements of plasmids with the *bla*_NDM-1_ gene were different: two IS*903* elements in pNDM-1_Dok01; two IS*26* elements in pNDM-HK; ΔIS*26* and ΔTN*3* in pKpANDM-1; and IS*Ec33* and IS*Sen4* in the plasmid of the *E. coli* 271 strain (Fig. 2). The *bla*_NDM-1_ gene in pNDM-1_Dok01 was flanked by IS*903*, suggesting that the gene was acquired as a composite transposon (Table 1).

**Table 1:** ORFs in NDM-1 composite transposon.

Gene_ID	Location	direction	gene	Top hit (blastp analysis)	Hit organism	aa identities
NDM1Dok01_N01630	129343..130266	+		gb|AAO15539.1| IS*903D* transposase	*Klebsiella pneumoniae*	307/307 (100%)
NDM1Dok01_N01640	130312..131013	-		ref|YP_025329.1| hypothetical protein pRA2_07	*Pseudomonas alcaligenes*	81/207 (39%)
NDM1Dok01_N01650	131197..131421	-		ref|ZP_04663571.1| hypothetical protein AbauAB_18243	*Acinetobacter baumannii* AB900	33/68 (49%)
NDM1Dok01_N01660	131543..132220	+		ref|YP_001966417.1| resolvase	*Moraxella bovis* Epp63	93/205 (45%)
NDM1Dok01_N01670	133044..133328	+		ref|ZP_06727037.1| acyltransferase	*Acinetobacter haemolyticus* ATCC 19194	70/82 (85%)
NDM1Dok01_N01680	133539..135068	-	*insE*	ref|YP_001102021.1| transposase InsE	*Salmonella enterica* subsp. *enterica* serovar Newport str. SL254	483/498 (97%)
NDM1Dok01_N01690	135257..136897	-	*groEL*	ref|YP_001102020.1| chaperonin GroEL	*Salmonella enterica* subsp. *enterica* serovar Newport str. SL254	489/533 (92%)
NDM1Dok01_N01700	136953..137243	-	*groES*	ref|YP_004145503.1| chaperonin Cpn10	*Pseudoxanthomonas suwonensis* 11-1	81/96 (84%)
NDM1Dok01_N01710	137437..137766	+		ref|YP_003374846.1| periplasmic divalent cation tolerance protein	*Xanthomonas albilineans* GPE PC73	64/100 (64%)
NDM1Dok01_N01720	137771..138802	+		ref|ZP_08267023.1| tat twin-arginine translocation pathway signal sequence domain protein	*Brevundimonas diminuta* ATCC 11568	141/188 (75%)
NDM1Dok01_N01730	138813..139451	-	*trpF*	gb|ADY00042.1| phosphoribosylanthranilate isomerase	*Escherichia coli* pNDM-HK	195/200 (98%)
NDM1Dok01_N01740	139456..139821	-		ref|ZP_05033688.1| glyoxalase family protein	*Brevundimonas* sp. BAL3	76/119 (64%)
NDM1Dok01_N01750	139825..140637	-	*bla*_NDM-1_	gb|ADP05158.1| New Delhi metallo-beta-lactamse 1	*Klebsiella pneumoniae*	270/270 (100%)
NDM1Dok01_N01760	140970..141893	-		ref|YP_961838.1| transposase, IS*4* family protein	*Shewanella* sp. W3-18-1	302/306 (99%)

The class I integron of pNDM-1_Dok01 is composed of the well-known integrase gene *intl1* and the antibiotic resistance markers *dfrA12*, *aadA2*, *qac-Δ1*, and *sul1* [3], [22], [23], while the integron in pNDM-HK shows only partial alignment with the *sul1* gene. In addition, the *bla*_TEM-1_ gene was identified in pNDM-1_Dok01 and pNDM-HK, but the adjacent regions were not found to be conserved between the plasmids. Overall, the variable region of these two plasmids was found to be composed of similar multiple antibiotic resistance markers and IS elements; however, these markers appear to exhibit a distinct gene organization between the plasmids.

The alignment shown in Fig. 2 indicates that variable IS elements appear to be linked to the *bla*_NDM-1_ gene and suggests that at least four types of gene cassettes are associated with the acquisition of carbapenem resistance through the dissemination of variable incompatibility groups between the plasmids described above.

### Possible linkage between *bla*_NDM-1_ and chaperonins

The likely NDM-1 composite transposon included the molecular chaperonin *groES* and *groEL* genes, which are involved in general stress responses (Fig. 2) [24]. These genes were also found in the IncA/C plasmids pAR060302 and pSN254 (Fig. 1) [20]. The GroEL amino acid sequence in pNDM-1_Dok01 shows 92% identity (489/533 amino acids) with GroEL in pAR060302 and pSN254. The *groES* and *groEL* genes in pAR060302 and pSN254 appeared to be integrated between the well-known class I integron genes *aacC* and *qacEΔ1*, while those in pNDM-1_Dok01 were found adjacent to the *bla*_NDM-1_ gene.

**Figure 3:**
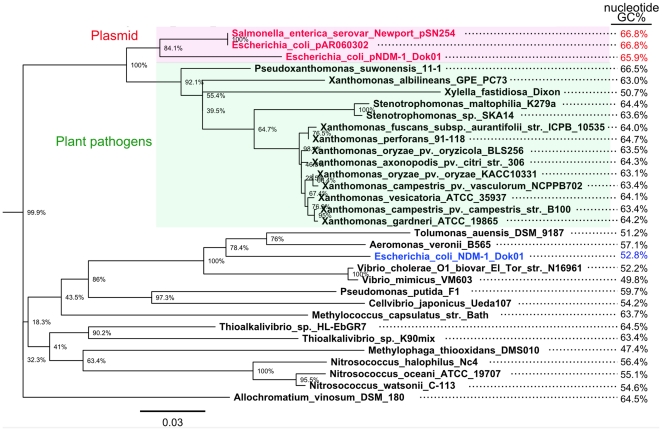
Phylogenetic tree of the whole amino acid sequences of chaperonin GroEL homologs. The amino acid sequences were selected and retrieved with a BLASTP search against the refseq_protein database with a cut-off value of 75% identity. The tree was constructed using the maximum likelihood method with 1,000 bootstrap replicates. The scale indicates that a branch length of 0.03 is 3 times as long as one that would show a 1% difference between the amino acid sequences at the beginning and end of the branch. The number at each branch node represents the bootstrapping value. The chromosomal GroEL in *E. coli* NDM-1_Dok01 is highlighted in blue. The GC percentage of the respective nucleotide sequences is shown on the right-hand side of the figure.

**Figure 4:**
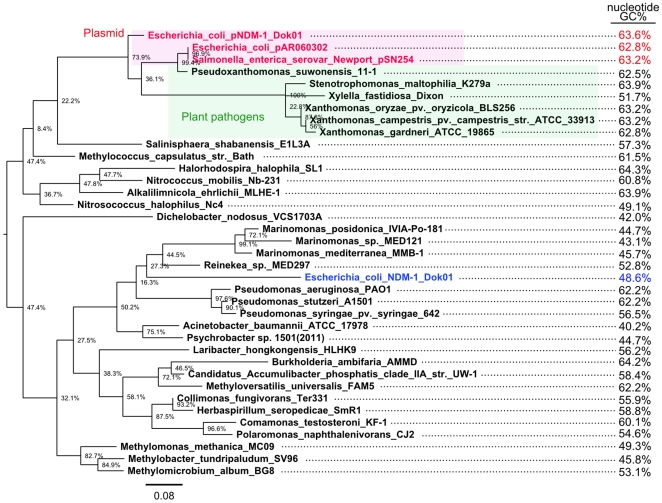
Phylogenetic tree of the whole amino acid sequences of chaperonin GroES homologs. Detailed analysis is same as Fig. 3.

Intriguingly, in addition to chromosomal chaperonin homologs, the additional acquisition of these chaperonin genes via the transposon could be used to predict their genetic source by horizontal gene transfer. In fact, phylogenetic analysis of the GroEL homologs suggests that the plasmid-derived GroEL proteins are similar to the homologs of the plant pathogens *Xanthomonas* and *Pseudoxanthomonas* spp. rather than to the chromosomal homologs of *E. coli* and other γ-proteobacteria (Fig. 3). Furthermore, the GC percentage of the putative *bla*_NDM-1_ transposon is remarkably higher than the other regions in pNDM-1_Dok01 (64.5% vs. 51.0%, respectively) (Fig. 1). The nucleotide sequence of *groEL* in pNDM-1_Dok01 had a higher GC of 65.9%, and an overall comparison indicated that among the characterized *groEL* homologs, the *Pseudoxanthomonas suwonensis* 11-1 (66.5%) had a GC percentage most similar to that of pNDM-1_Dok01 (Fig. 3). In addition to GroEL, GroES in pNDM-1_Dok01 had a high similarity (81/96 amino acids; 84% identity) to *Pseudoxanthomonas suwonensis* 11-1 (Table 1 and Fig. 4). Other CDSs in the putative *bla*_NDM-1_ transposon also showed high similarity with environmental bacteria such as *Pseudomonas, Acinetobacter, Xanthomonas,* and *Brevundimonas* spp. (Table 1).

## Discussion

The present study revealed the complete sequence of the plasmid pNDM-1_Dok01, which harbors the *bla*_NDM-1_ gene. Contrary to the IncL/M incompatibility plasmid pNDM-HK, pNDM-1_Dok01 belongs to the IncA/C incompatibility group. Similar to IncL/M plasmids, IncA/C plasmids are widely distributed among *Enterobacteriaceae*, including *Citrobacter freundii*, *Enterobacter cloacae*, *E. coli*, *Klebsiella pneumoniae*, *Proteus mirabilis*, *Salmonella enterica*, and *Serratia marcescens* [10]. Among IncA/C plasmids, pNDM-1_Dok01 showed a well-conserved plasmid structure with *E. coli* pAR060302 and *Salmonella* Newport pSN254, implying that the plasmid could be frequently transmitted among virulent *Enterobacteriaceae*. Indeed, a recent report revealed that variable length NDM-1-positive IncA/C plasmids were identified from two *E. coli* isolates, one *Vibrio cholerae* isolate, and one *Citrobacter freundii* isolate [8], suggesting that variable NDM-1-positive IncA/C plasmids have emerged in *Enterobacteriaceae*. Conversely, some NDM-1 plasmids such as *E. coli* p271A, could not be typed with the PBRT method [8], [21], indicating that the manner of their comprehensive transmission remains to be elucidated. In this study, whole sequencing of the plasmid was notably useful for replicon typing.

Further focusing on *E. coli* isolates, MLST analysis revealed that NDM-1_Dok01 can be classified as ST38 [7]; thus far, NDM-1 producing *E. coli* strains have been identified as ST11 [25], ST23 [25], ST101 [9], [26], [27], ST131 [28], [29], ST167 [9], and ST405 [30]. Although these observations suggest the widespread prevalence of the *bla*_NDM-1_ gene among various *E. coli* ST types, the NDM-1 producing *E. coli* ST38 type [7] appears to be a minor strain, thus far. Regarding the ST38 type, highly clonal *E. coli* ST38 type isolates (O86:H18) harboring the CTX-M-9 group *bla*_CTX-M_ spread throughout Japan as an epidemic strain over a short period of time during 2002–2003 [31]. In addition, ST38 was one of the epidemic strains isolated from community-onset urinary and intra-abdominal infections in the Netherlands [32]. ST38 appears to have virulence potential; indeed, the NDM-1_Dok01 strain showed serum resistance as a result of capsule synthesis from a small plasmid [33].

Regarding the acquisition of the *bla*_NDM-1_ gene, sequence alignment showed that variable IS elements could be associated with the transposition of the gene (Table 1 and Fig. 2). The *bla*_NDM-1_ gene in pNDM-1_Dok01 is flanked by two IS*903* elements, which are the terminal elements of the kanamycin resistance transposon Tn*903* (aminoglycoside-phosphotransferase-3′-I) [34]. The identification of such differential flanking terminal elements suggests that the *bla*_NDM-1_ gene has been widely transposed as a cassette gene with variable mobile elements.

A further intriguing finding was the acquisition of additional chaperonin genes, *groES* and *groEL,* in the *bla*_NDM-1_-related composite transposon (Table 1 and Fig. 2). This was not a result of the gene duplication of the chromosomal *groES* and *groEL* because phylogenetic analysis indicated that the additional homolog in pNDM-1_Dok01 was apparently related to those from other bacteria that are known to be plant pathogens such as *Pseudoxanthomonas*, *Xanthomonas*, and *Xylella* spp. In addition, the *groEL* homolog in pNDM-1_Dok01 had a higher GC percentage than the chromosomal homologs (GC: 52.8%), thereby providing additional support for the results from the homology search of the amino acid sequences.

Indeed, CTX-M chromosomal β-lactamase genes have been identified as potential sources of specific *bla*_CTX-M_ genes in different *Kluyvera* spp. [23], [35], [36]. Zheng *et al.* reported that NDM-1 had an amino acid identity of 55% with β-lactamase II from *Erythrobacter litoralis* [37]. *Erythrobacter* spp. are a putative source of NDM-1; however, a GroEL homology search to pNDM-1_Dok01 showed that the homolog in *Erythrobacter* had 66% less identity than that of *Pseudoxanthomonas*, implying that plant pathogens, such as *Pseudoxanthomonas* or related bacteria, could be a more likely source of the *bla*_NDM-1_ gene. Further comprehensive characterization of environmental bacteria will be required to elucidate the source and to show actual horizontal gene transfer.

These observations raise the question as to how multiple chaperonins contribute to fitness in variable conditions such as general stress or environment. To date, multiple chromosomal chaperonins have been identified in *Chlamydiae* and *Cyanobacteria* spp. [38]. *Chlamydiae* are obligate intracellular pathogens [39], and all known *Chlamydiae* can only grow by infecting eukaryotic host cells. Three paralogs of GroEL in *Chlamydiae* spp. are regulated under different conditions such as general stress or monocyte phagocytosis [38], suggesting that their acquisition might be beneficial for adaptation to variable stress conditions, including antibiotic selection.

In conclusion, the complete sequence of pNDM-1_Dok01 suggests that the *bla*_NDM-1_ gene was acquired by a novel composite transposon on an extensively disseminated IncA/C plasmid in the *E. coli* ST38 isolate. Further replicon typing and DNA sequencing of NDM-1-positive plasmids will be required to elucidate the extensive dissemination of these plasmids by horizontal gene transfer.
